# Bio-SimVerb and Bio-SimLex: wide-coverage evaluation sets of word similarity in biomedicine

**DOI:** 10.1186/s12859-018-2039-z

**Published:** 2018-02-05

**Authors:** Billy Chiu, Sampo Pyysalo, Ivan Vulić, Anna Korhonen

**Affiliations:** Language Technology Laboratory, DTAL, University of Cambridge, 9 West Road, Cambridge, CB39DB UK

**Keywords:** Word similarity, Intrinsic evaluation, Downstream tasks

## Abstract

**Background:**

Word representations support a variety of Natural Language Processing (NLP) tasks. The quality of these representations is typically assessed by comparing the distances in the induced vector spaces against human similarity judgements. Whereas comprehensive evaluation resources have recently been developed for the general domain, similar resources for biomedicine currently suffer from the lack of coverage, both in terms of word types included and with respect to the semantic distinctions. Notably, verbs have been excluded, although they are essential for the interpretation of biomedical language. Further, current resources do not discern between semantic similarity and semantic relatedness, although this has been proven as an important predictor of the usefulness of word representations and their performance in downstream applications.

**Results:**

We present two novel comprehensive resources targeting the evaluation of word representations in biomedicine. These resources, Bio-SimVerb and Bio-SimLex, address the previously mentioned problems, and can be used for evaluations of verb and noun representations respectively. In our experiments, we have computed the Pearson’s correlation between performances on intrinsic and extrinsic tasks using twelve popular state-of-the-art representation models (e.g. word2vec models). The intrinsic–extrinsic correlations using our datasets are notably higher than with previous intrinsic evaluation benchmarks such as UMNSRS and MayoSRS. In addition, when evaluating representation models for their abilities to capture verb and noun semantics individually, we show a considerable variation between performances across all models.

**Conclusion:**

Bio-SimVerb and Bio-SimLex enable intrinsic evaluation of word representations. This evaluation can serve as a predictor of performance on various downstream tasks in the biomedical domain. The results on Bio-SimVerb and Bio-SimLex using standard word representation models highlight the importance of developing dedicated evaluation resources for NLP in biomedicine for particular word classes (e.g. verbs). These are needed to identify the most accurate methods for learning class-specific representations. Bio-SimVerb and Bio-SimLex are publicly available.

## Background

Word representation models represent words in a continuous vector space so that semantically similar words obtain similar word representations. The vector spaces are typically induced from large unannotated corpora and serve as useful features for downstream Natural Language Processing (NLP) tasks [1, 2]. Recently, different representation models, such as Skip-gram (SG) and Continuous Bag of Words (CBOW) [3], have been proposed. They support a variety of important tasks in biomedical NLP, including Named Entity Recognition (NER) [4, 5] and text classification [6].

With the growing use of word representation models in NLP tasks, the quality and consistency of their evaluation have pivotal in their development [5, 7]. Existing evaluation protocols can be broadly categorised into two groups: *intrinsic* and *extrinsic*. A standard intrinsic evaluation protocol is the word similarity task: given a list of word pairs with different degrees of similarity, the task involves comparing **a)** the gold similarity ranking produced by humans, and **b)** the one computed automatically based on a representation model. The quality of the model is determined based on the Spearman’s correlation between its similarity ratings and the ratings assigned by human experts. On the other hand, an extrinsic evaluation protocol measures the quality of a representation model based on its performance in one or more actual (*downstream*) NLP tasks. Intrinsic evaluation is a computationally inexpensive method for measuring the quality of representation models. It facilitates the estimation of general properties of representation models, which relate to their task performance. As a consequence, it provides a practical means to compare models efficiently before applying them to more elaborate and computationally expensive extrinsic tasks.

While several wide-coverage intrinsic evaluation resources have been developed for the general domain (e.g. SimLex-999 [8] and SimVerb-3500 [9]), existing resources for biomedicine such as UMNSRS [10] and MayoSRS [11] suffer from notable shortcomings. First, they fail to distinguish between the concepts of semantic similarity (e.g. *dyspnea* and *tachypnea*) versus semantic relatedness (e.g. *pneumonia* and *infiltrate*). With current datasets, models which capture the fact that *pneumonia* and *infiltrate* are dissimilar get penalised: we analyse this discrepancy in “Evaluation resources in the biomedical domain” section. Recent research shows that such distinctions are important predictors concerning the usefulness of representation models in extrinsic tasks such as NER and part-of-speech tagging [5, 7].

Second, recent research has demonstrated that developing different learning approaches for individual word classes can greatly extend the usefulness of representation models [12, 13]. However, there is no standard scheme in biomedical NLP tailored for the intrinsic evaluation of representation models for prominent word classes such as verbs. Verbs constitute an integral part of a sentence, and consequently human communication. Many NLP tasks, including relation extraction (see e.g. Nguyen et al. [14]), use the syntactic structure of verbs (i.e. the predicate-argument structure) to identify relations in biomedical text. Moreover, a broad range of verbs (e.g., *attach*, *bind* and *interact*) can refer to the same relations (e.g. protein-protein interactions). An accurate representation model which takes into account complex syntactic-semantic properties of verbs is crucial for such biomedical systems to succeed in identifying relations between entities described in text. A reliable metric that can compare different representation models for biomedical verbs would facilitate the development of such systems. However, current benchmarks used in the biomedical domain (UMNSRS, MayoSRS) evaluate nouns only.

To tackle these issues, we introduce two novel resources for the intrinsic evaluation of noun and verb representations in the biomedical domain: *Bio-SimLex* and *Bio-Simverb*, which are unprecedented in both size and coverage. They include 1454 noun types and 1,131 verb types respectively, sourced from 14 Open Biomedical Ontologies [15] and 14,823 journals in the PubMed Central Open Access subset (PMC), covering over 120 areas of biomedicine (counted by Broad Subject Terms, details in “Choice of words” section). The wide coverage allows researchers in different biomedical sub-domains to compare representation models easily. Furthermore, these resources have been developed using the established SimLex and SimVerb style annotation, which explicitly distinguishes between semantic similarity and relatedness. We report a positive correlation between the performance of various representation models on our intrinsic resources and extrinsic tasks. Consequently, these new resources may be used to estimate the effect of hyper-parameter tuning for different representation models, which plays a key role in achieving strong performance in extrinsic tasks. Finally, researchers are now able to evaluate noun and verb representations separately: this should facilitate and improve our understanding of how representations for particular word classes contribute to extrinsic tasks.

In the next section, we describe four standard intrinsic benchmarks used in the biomedical and general language domains, followed by the design and sampling procedure for our datasets in “Construction and content” section. We conduct a detailed analysis of the inter-annotator agreement (IAA, in Spearman’s *ρ*): our datasets obtain moderate to high IAA (0.65≤*ρ*≤0.78) with twelve annotators. We also compare the ranking of representation models on our datasets and other benchmarks with their ranking in downstream applications, relying on four extrinsic tasks (details in “Experimental setup” and “Utility and discussion” sections). For twelve off-the-shelf representation models (details in “Experimental setup” section), we obtain positive correlations between the two sets of scores. In “Subset evaluation” section, we demonstrate how to use Bio-SimVerb and Bio-SimLex for different linguistic analyses, previously impossible due to the lack of coverage and scale in existing resources. The resources are publicly available to the research community at: https://github.com/cambridgeltl/bio-simverb.

## Related work

### Evaluation resources in general domain

The creation of intrinsic resources in NLP is mostly limited to the general domain, with a range of datasets created over the years. For instance, RG-65 [16] and MC-30 [17] are small-scale datasets (65 and 30 word pairs correspondingly) in the general domain which evaluate only noun representations. There are also datasets, such as YP-130 (130 word pairs), which only evaluate verb representations. Larger datasets, such as MTurk-287 [18] and MTurk-771 [19], have been constructed by crowdsourcing human similarity ratings using Amazon Mechanical Turk. Further, WS-353 [20] contains 353 English word pairs with similarity ratings also provided by human annotators. WS-353 is further divided into two groups which evaluate relatedness and similarity separately [21]. Rare-Words [22] is a dataset which focuses on the evaluation of low-frequency words.

Chiu et al. [7] report a negative correlation between the performance of various representation models on all these datasets and extrinsic tasks. The only exception is SimLex-999 (referred to as SimLex henceforth, [8]). Following an identical sampling procedure, SimVerb-3500 (SimVerb, [9]) may be seen as an extension of SimLex that emphasises a high-quality evaluation of verb similarity. SimVerb expands the coverage of distinct verb types from 222 in SimLex to 837, covering all verb classes represented in VerbNet [23, 24]. SimLex and SimVerb consist of 999 and 3500 word pairs (resp.) rated by humans for true semantic similarity instead of a broader notion of (conceptual) semantic relatedness [8]. These ratings are used to compare against the ratings produced by various representation models computed by cosine similarity between the two words forming a pair. The quality of a representation model is determined based on the Spearman’s correlation between its similarity ratings and the ratings assigned by human experts to the word pairs. When compared with other datasets of similar nature, SimLex and SimVerb use a different rating principle. In the next section, we will describe this principle and compare it with the ones used in datasets in biomedicine.

### Evaluation resources in the biomedical domain

MayoSRS [11] and UMNSRS [10] are two intrinsic evaluation benchmarks in the biomedical domain. MayoSRS consists of 101 clinical term pairs, which are generated manually by a physician. The relatedness of each word pair is rated by nine medical coders and three physicians based on a ten-point scale (1: closely related, 10: unrelated). UMNSRS consists of 566 and 587 medical word pairs for measuring semantic relatedness. Word pairs included in the dataset are sourced by first selecting all concepts from the Unified Medical Language System (UMLS, [25]) with one of three semantic types: disorders, symptoms and drugs, followed by a manual filtering from a physician. The degree of association between terms in each data set is then rated by four medical residents from the University of Minnesota Medical School.

In terms of size and coverage, MayoSRS is smaller and puts focus on clinical concepts, whereas UMNSRS covers more concepts from different areas of biomedicine (e.g. drugs and disorders). Both datasets include multi-word expressions (e.g., “difficulty walking”, “aloe vera”). Both resources cover only nouns, and they do not extend to other important classes of words, such as verbs.

When comparing SimLex and SimVerb with UMNSRS and MayoSRS, a fundamental distinction is their annotation for word similarity. UMNSRS considers related words as similar whereas SimLex and SimVerb consider related words as dissimilar (e.g. *coffee* and *cup*). Thus, in their annotation procedures, participants are instructed to give low scores for related but dissimilar word pairs (e.g. *bell* and *door*). In contrast, MayoSRS only considers word relatedness. Hence, there are cases where related but semantically dissimilar word pairs (e.g. *pneumonia* and *infiltrate*) are rated higher than those that are both related and similar (e.g. *dyspnea* and *tachypnea*). Consequently, evaluation of representation models on these datasets penalises the models which capture the fact that *pneumonia* and *infiltrate* are dissimilar.

As mentioned, Chiu et al. [7] compare SimLex with other datasets which do not separate the evaluation of similarity and relatedness. They report a higher correlation between intrinsic and extrinsic scores with SimLex and suggest that individual tasks require different types of semantic similarity. For example, if the task is POS tagging, *pneumonia* and *malnutrition* should be considered as instances of the same equivalence class (i.e., nouns) by the model even though they are not semantically similar. In contrast, semantic similarity between entities such as co-hyponymy (e.g. *Italy* and *Spain* are co-hyponyms of the hypernym *Country*.) is essential for NER or human language understanding tasks such as dialogue [26]. Hence, separating the evaluation of similarity and relatedness allows for fine-grained estimation of such task-specific similarity.

## Construction and content

### Choice of words

Samples/words in Bio-SimVerb (verbs) and Bio-SimLex (nouns) are collected from a pre-processed PubMed Central Open Access subset (PMC), which is distributed by Hakala et al. [27]. POS tags and tokens in this resource are generated using the BLLIP constituency parser [28], trained on a biomedical corpus [29]. The resource covers over 1.4M full articles with more than 388M parsed sentences.

After retrieving all samples from the PMC, we remove all multi-word expressions (e.g. “37 degrees C”) and auxiliary verbs (e.g. “must”). We also filter out noise, such as symbols (e.g. “ <"), numbers (e.g. “2010”), strings too short to be reliably understood (e.g. “a”, “v”, “b1”) and Greek letters (“ *α*”). In the next step, we use the Bio-lemmatizer [30] for lemmatisation of non-lemmas (e.g. “gone”, “went”, “cells”). We also normalise words with the British English spelling into their American English variants for consistency. We exclude terms occurring less than five times, as they are most likely uninformative. These steps filter down our samples from 20,281 to 6425 verbs, and from 1,339,806 to 217,425 nouns. We have then invited two researchers working in biomedical NLP to determine whether these terms are mostly used in the biomedical or general domains. We exclude samples with ambiguous and frequent usage in both domains (e.g. “play”, “fire”). Consequently, 526 and 483 verbs, plus 1312 and 840 nouns, are categorised as commonly used in the biomedical domain and general domain, respectively. Several example words from both domains are provided in Table 1.

**Table 1:** Biomedical- and general-domain word samples in Bio-SimVerb and Bio-SimLex

Biomedical	General
Depolymerize	Automate
Electrophorese	Study
Phosphorylate	Argue
Centrosome	Idea
Pathophysiology	People
Endothelium	River

To show that the selected biomedical terms are domain-specific, we have examined individual samples based on their frequency differences in the biomedical and general English texts. We compare the relative frequency of our samples in PMC with that in the British National Corpus (BNC) [31]. We calculate the Spearman’s correlation (*ρ*) between their frequency ranking in these corpora. The result is only a weak correlation: *ρ* = 0.39, implying that the usage patterns of words in these areas are distinct.

To ensure a broad coverage of samples from various areas of biomedicine, we keep track of every journal where a sample appears. These journals are categorised by 125 Broad Subject Terms [32], which are assigned by the U.S National Library of Medicine (NLM) to MEDLINE journals in order to describe the journal’s overall scope and nature. For each sample obtained from PMC, we record the PMCIDs of all the journals in which it appears. We then map the PMCIDs to their corresponding Broad Subject Terms. Consequently, we generate the distribution of Broad Subject Terms for individual samples based on their occurrence in journals. Since one sample can appear in journals with different Broad Subject Terms, we assign the one with the highest occurrence frequency.

The use of Broad Subjects Terms and the examination of frequency for our samples demonstrate the extensive coverage of words in Bio-SimLex and Bio-SimVerb originating from different biomedical areas.

### Constructing concept pairs

Next, we sketch the process of constructing concept word pairs for the final annotation. In general, our dataset is made up of *quarters* of word pairs: around 250 associated pairs and 250 unassociated pairs are from the biomedical domain; 250 associated pairs and 250 unassociated pairs are from the general domain.

#### Concept pairs from the biomedical domain

To form associated pairs in the biomedical quarter, we use two publicly available semantic resources:

**Specialist Lexicon:** a part of the Unified Medical Language System (UMLS), the SPECIALIST Lexicon provides information about common English vocabulary and biomedical terms found in MEDLINE as well as in the UMLS Metathesaurus. Each entry in SPECIALIST includes syntactic (e.g. *form* and *forms*), morphological (e.g. *localised* and *localized*), and semantic variants (e.g. *breathe* and *respire*). To form associated pairs, we pair up our concepts randomly sampled from the PMC. From these random pairings, we have detected that 121 noun and 80 verb synonymous pairs appear in SPECIALIST. These pairs, together with pairs found in other resources (described in the next section), are included in Bio-SimLex and Bio-SimVerb after a manual inspection by our biomedical NLP researchers.

**The Open Biomedical Ontologies:** The Open Biomedical Ontologies Foundry [15] creates a collection of ontologies for shared use across different biological and medical domains. Each ontology provides a fine-grained representation of similar entities within a sub-domain. We use synonymous, as well as sibling entities (i.e., entities sharing the same parent node in an ontology), provided in 14 ontologies (see Table 2) as the reference for finding synonymous pairs. Since many terms in these ontologies are nominalised forms of verbs (e.g. *phosphorylation* instead of *phosphorylate*), we first include all word forms for every term in the Ontologies by querying its morphological variants in the SPECIALIST Lexicon. Following that, we match our random pairs to the synonymous pairs found in these ontologies.

**Table 2:** Fourteen Ontologies used for sampling synonymous pairs in Bio-SimVerb and Bio-SimLex

Ontology	Reference
Chemical Entities of Biological Interest (ChEBI)	[53]
Gene Ontology (GO)	[54, 55]
NCI Thesaurus (NCIT)	[56]
Foundational Model of Anatomy (FMA)	[57]
Disease Ontology (DOID)	[58]
Uberon multi-species anatomy ontology (UBERON)	[59, 60]
Plant Ontology (PO)	[61, 62]
Plant Phenotypes and Traits (PATO)	[63]
Ontology for Biomedical Investigations(OBI)	[64]
Molecular Process Ontology (MOP)	[65]
Zebrafish anatomy and development (ZFA)	[66]
Protein modification (PSI-MOD)	[67]
Common Anatomy Reference Ontology (CARO)	[68]
Xenopus anatomy and development (XAO)	[69, 70]

From our random pairs, we find 506 (nouns) and 287 (verbs) synonymous pairs in these ontologies, together with the semantic pairs previously found in SPECIALIST (nouns: 121 and verbs: 80). This yields a total of 627 noun pairs and 367 verb pairs. They are all inspected by our biomedical NLP researchers manually to ensure that pairs are associated in a biomedical sense. The experts agree that 247 noun pairs and 250 verb pairs have an association: this forms the quarter of associated word pairs in the biomedical domain.

Using a set of random pairs which are not found in any of the two semantic resources, we randomly sample 247 noun pairs and 250 verb pairs. They form the quarter of unassociated pairs in the biomedical domain.

#### Concept pairs from the general domain

Bio-SimLex and Bio-SimVerb contain 494 noun pairs and 500 verb pairs that are commonly used in general English. We now describe how to form such word pairs from our samples, with reference to the USF norms data set [33] containing word association norms.

**The USF norms dataset:** The USF data set is the largest database of free word association collected in word norming experiments for English. It comprises 72,000 associated word pairs. The pairs are created by presenting one of 5000 cue concepts to human subjects, and then recording their first associated words. This way, each concept is rated by over 10 participants, yielding a set of associates for every concept. In addition, the forward and backward association strengths between a concept and its associates are reported in the USF. The USF includes both related but dissimilar pairs (e.g. *player/team*), as well as similar pairs (e.g. *to wash/to rinse*).

In our case, we again pair up concepts randomly sampled from the PMC. From these pairs, we extract 247 noun pairs and 250 verb pairs represented in the USF: we require the pairs to be assessed by more than 10 USF participants, as well as to have both forward and backward association strengths assigned. These two filtering conditions not only ensure that two words in a pair have a degree of semantic association but also guarantee that the association link is bidirectional. A similar sampling procedure is used in the construction of general-domain benchmarks including SimLex [8] and SimVerb [9]. Finally, we also extract 247 noun pairs and 250 verb pairs not present in the USF to form the quarter of unassociated words pairs in the general domain.

### Concept pair scoring

Bio-SimLex and Bio-SimVerb consist of 988 noun pairs and 1000 verb pairs respectively. Similarity between concepts in each pair is determined by twelve annotators who all have a background in biology. Seven annotators are undergraduate or post-graduate students in the Biology School, University of Cambridge, while the remaining five are biologists working at the Institute of Environmental Medicine, Karolinska Institutet. The similarity is assessed on a scale of 0-6, where 0 is assigned to completely unrelated concepts and 6 represents highly synonymous concepts. The same scale is used in the construction of SimVerb and SimLex.

We adopt the annotation protocol established in prior work on SimVerb and SimLex: the annotators are instructed to assign low scores to related but dissimilar word pairs (e.g. *drug/pharmacy*). In each data set, we randomly select 50 pairs to serve as a consistency set. This set is used to detect possible variation between annotators and data subsets. We then divide all pairs from Bio-SimVerb and Bio-SimLex into two groups, containing approximately 600 pairs each. Out of these 600 pairs, 500 are unique to each group, and 50 pairs are from the consistency set, included in both groups. Another 50 are duplicate pairs displayed to each rater twice to detect his or her inconsistent annotations. Each annotator rates one group. Consequently, each pair is rated by six participants in total. The final survey is implemented so that each rater sees 120 pairs per page on the interface: 100 unique ones, 10 from the consistency set, and 10 duplicate pairs.

The pairs are rated by moving a slider. The participants are explicitly asked to give the same rating to same pairs for consistency. Furthermore, we also monitor for suspicious rating patterns (e.g., randomly alternating between two ratings). If a participant uses a single rating for ten consecutive questions, we issue a warning to the participant as a reminder to pay attention throughout the survey.

## Experimental setup

### Word representation models

To evaluate Bio-SimVerb and Bio-SimLex, we apply a range of popular word representation models. All models are trained on a corpus of PubMed abstracts consisting of approximately 2.7 billion tokens (11,980,338 types). The common hyper-parameters shared by these models are standardised to the values shown in Table 3, while parameters specific to individual models are kept at their defaults.

**Table 3:** Hyper-parameter values for word representation models. Parameters specific to individual models are set to their defaults

Parameters	Values
Context window size	5
Vector dimension	200
Learning rate	0.05
Negative sampling	5
Min-count	5
Sampling rate	1e-5

**Skip-Gram (SG) and Continuous Bag of Words (CBOW)** The word2vec tool [3] has been shown to produce highly competitive representation models in many intrinsic and extrinsic tasks [4, 6, 34, 35], as compared to models such as Random Indexing [36] and Latent Semantic Analysis [37], among others. In particular, Muneeb et al. [38] show that SG models outperform models such as GloVe [39] on word-similarity tasks. Hence, the representation models used in these experiments are mostly built on the SG and CBOW architectures. In the SG model, the vector for each word is learned by predicting other words within a given context window. Conversely, in the CBOW model, a word is predicted given its context.

**Structured Skip-Gram (SSG)** Based on the SG model, Ling et al. [40] proposed an extension, Structured Skip-Gram (SSG), which captures word order information. In the SSG model, the vector of each word is learned by predicting not only its context words, but also their relative position. This model has shown improvement in various syntactic tasks as compared to original SG models [40].

**CBOW with attention (Attention)** Based on the CBOW architecture, Ling et al. [41] introduced an attention mechanism which finds the contextual words that are most relevant for each prediction. Their results showed that this model can benefit both semantic and syntactic tasks [41].

**SG with dependency-parse (Dependency)** Levy et al. [42] proposed using dependency-parsed texts to help representation learning in word2vec, so that learning includes syntactic dependencies and is not restricted to a fixed context window. This model has been shown to better capture the functional similarity of words than the original SG models [42].

In addition to applying the above models, we also include seven previously released word representations:

**PubMed-w2v and BioASQ** created by Pyysalo et al. [4] and Kosmopoulos et al. [43] (resp.) and built with the SG model with vector dimension of 200 and a context window size of 5.

**Paragram, Paragram+CF, Symmetric, CBOW-general and Dep-general**

Biomedical representation models are domain-specific, which imply that the word semantics they capture can be different from those in the general domain. To study this, we also include five general-domain representation models previously benchmarked on SimVerb and SimLex: a model learned from the paraphrase database (**Paragram**, [44]) and its extension fine-tuned by linguistic constraints from other knowledge resources (**Paragram+CF**, [45]), a model learned from symmetric-patterns in corpus such as “x rather than y” and “either x or y” (**Symmetric**, [12]) as well as CBOW (**CBOW-general**) and dependency models (**Dep-general**).

### Intrinsic evaluation

We perform intrinsic evaluations on the benchmarks described in “Related work” section. We use the standard experimental protocol for word similarity tasks: for each word pair in a dataset, we compute the cosine similarity of the two word representations and rank the word pairs by these values. We then compare the ranking against a ranking based on human similarity scores using Spearman’s correlation (*ρ*).

### Extrinsic evaluation

We assess our representation models using a NER task with four established corpora: the Anatomical Entity Mention corpus (AnatEM) [46], the BioCreative II Gene Mention task corpus (BC2GM) [47], the BioCreative IV Chemical and Drug NER corpus (BC4CHEMD) and the JNLPBA corpus (JNLPBA) [48].

The NER model follows the simple window-based feed-forward network architecture proposed by Collobert et al. [49]. Table 4 shows the hyper-parameters used in this model. The model input consists of the vectors of words within a context window, connected to a single hidden layer with a hard tanh activation, leading to an output Softmax layer for predicting labels for named entities. Performance is evaluated using entity-level *F*-score as implemented in the standard conlleval evaluation script.

**Table 4:** Hyper-parameters used in NER

Parameters	Values
Vector dimension	200
Hidden layer dimension	300
Context window size	5
Learning rate	0.01
Dropout probability	0.2
Epochs	20
Minibatch size	50

## Utility and discussion

### Inter-rater reliability

In this study, each annotator rated one sub-group of pairs in Bio-SimVerb and Bio-SimLex. We used the previously published implementation from the SimLex and SimVerb studies to estimate inter-annotator agreement (IAA). In this implementation, IAA-1 computes the average pairwise Spearman’s correlation (*ρ*) of ratings for each annotator with the ratings of all the other annotators. To smooth individual rater effects, we also include IAA-2 (mean), which computes the Spearman’s correlation (*ρ*) of individual annotators’ ratings with the average ratings of all the other annotators within the same group.

We first computed IAA-1 between the ratings of all annotators on the consistency set. Based on these results, we removed from the data the annotations of one outlier whose IAA-1 was considerably lower than the average IAA-1 of all the other annotators. After that, we computed IAA-1 and IAA-2 between annotators rating the same group. The average IAA-1 and IAA-2 for Bio-SimVerb are 0.65 and 0.69 respectively, whereas the results for Bio-SimLex are 0.72 (IAA-1) and 0.78 (IAA-2). We then calculated the average of all ratings from the accepted annotators for each pair, and scaled the scores linearly from the 0–6 to the 0–10 interval to match other datasets such as MayoSRS. To apply the resulting resources, the similarity score for a representation model is computed using cosine similarity for each word pair, and the performance of the model is then measured by the Spearman’s correlation between its ranking of the pairs and the human ranking.

### Performance of representation models on intrinsic evaluation datasets

Table 5 shows the intrinsic (left 5 columns) and extrinsic scores (right 4 columns) of the different representation models. To address ties in human scores in intrinsic evaluations, we use the Scipy implementation [50] to compute the tie-corrected Spearman’s correlation as suggested by Kendall et al. [51]. This correction handles the ties by averaging the uncorrected correlation values over all possible valid (without ties) rankings of the underlying variable. To account for variance in neural networks due to their random initialisation, we run three trials for all extrinsic tasks and report their averages. In general, scores are higher in Bio-SimLex than in Bio-SimVerb for all representation models, indicating that it is still difficult for current models to capture verb semantics. In particular, the score of the dependency model is low in Bio-SimVerb. This implies that using dependency parses to reach beyond bag-of-word context may not contribute equally to the representation learning of verbs and nouns. To a large extent, to identify learning algorithms that are useful for learning word-type specific representations, resources for the evaluation of specific word-types are a necessity.

**Table 5:** Intrinsic (left 5 columns) and extrinsic scores (right 4 columns) of different representation models trained on the biomedical corpus

	UMN-rel(*ρ*)	UMN-sim(*ρ*)	MayoSRS(*ρ*)	Bio-SimVerb(*ρ*)	Bio-SimLex(*ρ*)	BC4CHEMD (F-score)	BC2GM (F-score)	AnatEM (F-score)	JNLPBA (F-score)
Attention	0.5248	0.5551	**0.6113**	0.471	0.7155	79.11	65.91	80.49	62.3
SSG	0.5189	0.552	0.6003	**0.4744**	0.7181	79.62	67.3	81.3	63.78
SG	**0.5767**	**0.6271**	0.5744	0.4638	0.7151	81.37	70.2	81.32	65.16
CBOW	0.5	0.5348	0.5146	0.4367	0.702	78.41	64.05	80.3	61.9
Dependency	0.3934	0.4622	0.3445	0.3978	**0.7436**	**83.69**	**71.43**	**82.4**	65.01
PubMed-w2v	0.506	0.549	0.5133	0.4376	0.6984	80.71	67.4	81.1	64.86
BioASQ	0.5092	0.5893	0.4729	0.4228	0.6982	56.95	48.86	53.34	50.51

The bolded text implies the best performing models of their kind

### Correlation between intrinsic and extrinsic scores

From Table 5, we observe that there is variation in the performance of different representation models across different tasks. For example, the best-performing model in MayoSRS is the attention model, whereas the dependency model performs best in most NER tasks. To study if our datasets can predict extrinsic performance, we compute the Pearson’s correlation (*r*) to quantify the linear relationship between the intrinsic (UMNSRS, MayoSRS, Bio-SimVerb and Bio-SimLex) and the extrinsic scores (BC4CHEMD, BC2GM, AnatEM and JNLPBA).

Table 6 shows the correlation between the performances of representation models on various intrinsic evaluation datasets and the NER tasks. When compared to different benchmarks, the correlations between our datasets and downstream tasks are on par with or notably higher than the ones in UMNSRS and MayoSRS. The result suggests that our datasets can better predict the performance in NER, as compared with other intrinsic evaluation standards in biomedical NLP. Nevertheless, we find that there is no statistically significant correlation on any dataset (two-tailed t-test with *alpha* = 0.05). A possible reason is that the experiment involves only a limited number of data points, and only very large effects can be statistically significant.

**Table 6:** Pearson’s correlation between word-similarity/Bio-SimVerb and Bio-SimLex scores and the NER tasks evaluated on biomedical representation models trained with different approaches. None of the scores are statistically significant

	BC4CHEMD	BC2GM	AnatEM	JNLPBA
UMN-rel	-0.15	-0.14	-0.08	-0.07
UMN-sim	-0.38	-0.34	-0.34	-0.3
MayoSRS	0.08	0.04	0.18	0.12
Bio-SimVerb	0.2	0.18	0.29	0.24
Bio-SimLex	**0.53**	**0.6**	**0.46**	**0.48**

Bold: best scores

Next, we compute the same performance-correlations using a set of SG models with different context window sizes (other hyper-parameters are kept default). The scores for individual tasks and their correlations are shown in Tables 7 and 8 respectively.

**Table 7:** Intrinsic (left 5 columns) and extrinsic scores (right 4 columns) of the biomedical representation models trained using different window sizes

Window Size	UMN-rel(*ρ*)	UMN-sim(*ρ*)	MayoSRS (*ρ*)	Bio-SimVerb(*ρ*)	Bio-SimLex(*ρ*)	BC4CHECMD (F-score)	BC2GM (F-score)	AnatEM (F-score)	JNLPBA (F-score)
1	0.5317	0.5759	0.5551	0.4594	**0.7294**	**81.51**	70.06	82.16	65.34
2	0.563	0.6144	0.6238	0.4696	0.7207	81.44	70	**82.21**	65.51
4	0.5768	0.6247	0.581	0.464	0.7188	81.5	70.04	82	65.75
5	0.5767	0.6271	0.5744	0.4638	0.7151	81.37	**70.20**	81.32	65.16
8	0.582	0.6377	0.5975	0.4611	0.7086	81.24	69.56	80.99	65.53
16	0.5888	0.6431	0.6123	0.4667	0.7034	81.02	69.39	80.72	64.78
20	0.5896	0.6418	0.6319	0.4584	0.7031	81.12	69.62	80.49	65.19
25	**0.6018**	**0.6489**	0.6188	0.4519	0.7004	81.07	69.93	80.92	65.14
30	0.6007	0.6457	**0.6486**	0.4502	0.7043	80.71	69.2	81.03	64.79

The bolded text implies the best performing models of their kind

**Table 8:** Pearson’s correlation between word-similarity/Bio-SimVerb and Bio-SimLex scores and the NER tasks evaluated on biomedical representation models trained with different window sizes

	BC4CHEMD	BC2GM	AnatEM	JNLPBA
UMN-rel	-0.78^a^	-0.56	-0.78^a^	-0.46
UMN-sim	-0.73	-0.57^a^	-0.81	-0.42^a^
MayoSRS	-0.78	-0.69	-0.54^a^	-0.47^a^
Bio-SimVerb	0.63	0.36	0.42	0.40
Bio-SimLex	**0.83** ^a^	**0.66**	**0.92** ^a^	**0.59**

Bold: best scores

^a^Statistically significant

With the same model architecture but different context window sizes, most extrinsic scores (right 4 columns of Table 7) have a performance peak with a narrow window (e.g. win= 1), followed by a gradual decrease when window size increases. The results in Table 8 show that our evaluation scores correlate better with downstream tasks than all other available intrinsic evaluation datasets. Although we only test on nine models, we observe two significant positive correlations in Bio-SimLex (BC4CHEMD and AnatEM). Notably, UMNSRS and MayoSRS show a negative correlation with all NER tasks. Similar patterns are previously reported by Chiu et al. [7] when comparing these scores using representation models trained with other corpora including PMC. They suggest that datasets such as MayoSRS emphasise modelling topical relatedness rather than similarity, which is learned better by a representation model with a larger context window. Nevertheless, tasks such as NER rely more on the modelling of similarity such as co-hyponymy, which is typically captured better with a narrow context window [52]. This disagreement in emphasis may lead to negative correlations between the intrinsic and extrinsic scores, as shown in Table 8. By contrast, we emphasised modelling relatedness and similarity separately during the annotation phase of Bio-SimLex and Bio-SimVerb. Annotators were instructed (with clear case examples) to give low scores to related but dissimilar word pairs, and this design lead to higher correlation with extrinsic tasks in our experiments. Our datasets thus capture some properties of word similarity and relatedness that can predict performance at extrinsic tasks. Further, Bio-SimLex shows a better correlation with extrinsic performance than Bio-SimVerb. One possible reason for this is that the extrinsic tasks we considered in this experiment are NER, where performance is closely related to the quality of noun representations. More importantly, these results confirm our hypothesis that evaluating the qualities of the representation models separately for various word types provides insight into how they individually contribute to extrinsic performance.

### Comparison with general-domain datasets

We have shown that our resources capture some properties (e.g. word semantics) that can predict performance in biomedical NER. These properties are expected to be domain-dependent, which suggests that it should be more effective to evaluate with in-domain datasets to predict performance for biomedical tasks. To study this, we use five representation models (detailed in “Word representation models” section), benchmarked on general-domain datasets (SimVerb and SimLex), and evaluate their performance-correlation on our datasets and biomedical tasks.

Table 9 shows the correlation between intrinsic and extrinsic scores for general-domain representation models. Most scores for general-domain datasets (SimLex and SimVerb) correlate negatively with biomedical NER tasks. Due to domain-specificity, the properties that SimVerb and SimLex measure generally do not reflect how well a representation model will perform in biomedical tasks, and may even give contradictory indications. Bio-SimLex achieves the best results also in this evaluation and shows a positive correlation with performance in BC2GM and JNLPBA despite measuring out-of-domain representation models. (In interpreting these results, it should be noted that none reaches statistical significance.)

**Table 9:** Pearson’s correlation between general-domain datasets/Bio-SimVerb and Bio-SimLex scores and the NER tasks evaluated on general-domain representation models benchmarked in SimVerb and SimLex. None of the scores are statistically significant

	BC4CHEMD	BC2GM	AnatEM	JNLPBA
SimVerb	-0.31	-0.09	-0.41	-0.12
SimLex	-0.36	-0.20	-0.49	-0.19
Bio-SimVerb	-0.38	-0.18	-0.47	-0.22
Bio-SimLex	**0.00**	**0.23**	**-0.09**	**0.18**

Bold: best scores

To summarise, Bio-SimVerb and Bio-SimLex are better predictors of performance in biomedical NER than other in-domain datasets (UMNSRS, MayoSRS) and general-domain datasets (SimLex, SimVerb). We observe moderate to high positive correlations between performance on our datasets and in biomedical NER, which are consistent across corpora and different models as well as within the same model architecture with different windows sizes. Although it is possible to use our datasets to evaluate general-domain representation models, the results indicate that they are most effective in the evaluation of biomedical domain representation models.

### Subset evaluation

The extensive coverage and scale of Bio-SimVerb and Bio-SimLex enable model evaluation based on various criteria. In this section, we showcase two examples.

**Fig. 1:**
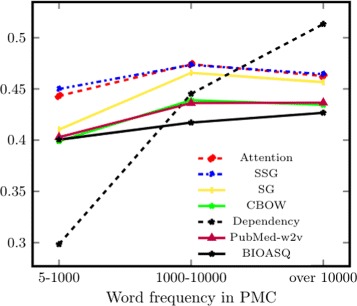
Subset-based evaluation for Bio-SimVerb (y axis unit: *ρ*), where subsets are created based on the word-frequency in PMC. To be included in each group it is required that both words in a pair are in the same frequency interval (x axis)

**Fig. 2:**
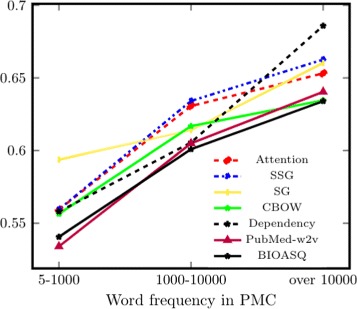
Subset-based evaluation for Bio-SimLex (y axis unit: *ρ*), where subsets are created based on the word-frequency in PMC. To be included in each group it is required that both words in a pair are in the same frequency interval (x axis)

**Frequency** We first select word pairs based on their frequency of occurrence in PMC and form three groups, with 300–400 pairs in each group. Results for Bio-SimVerb and Bio-SimLex are shown in Figs. 1 and 2 respectively. They suggest that the performance of all models improves as the frequency of the words in the pair increases. Since distributional models are data-driven, their qualities of capturing word-semantics are mainly governed by the word-frequency in the corpus.

**Broad subject terms** In general, words with more diverse usage patterns are expected to be harder to learn with statistical models. To test this hypothesis, we divide the word pairs into three groups based on their numbers of Broad Subject Terms, which represent the sub-domains of text in which a word appears. Words that have more Broad Subject Terms appear in text across different areas of biomedicine and tend to have more diverse usage patterns compared to words used only in a single domain.

**Fig. 3:**
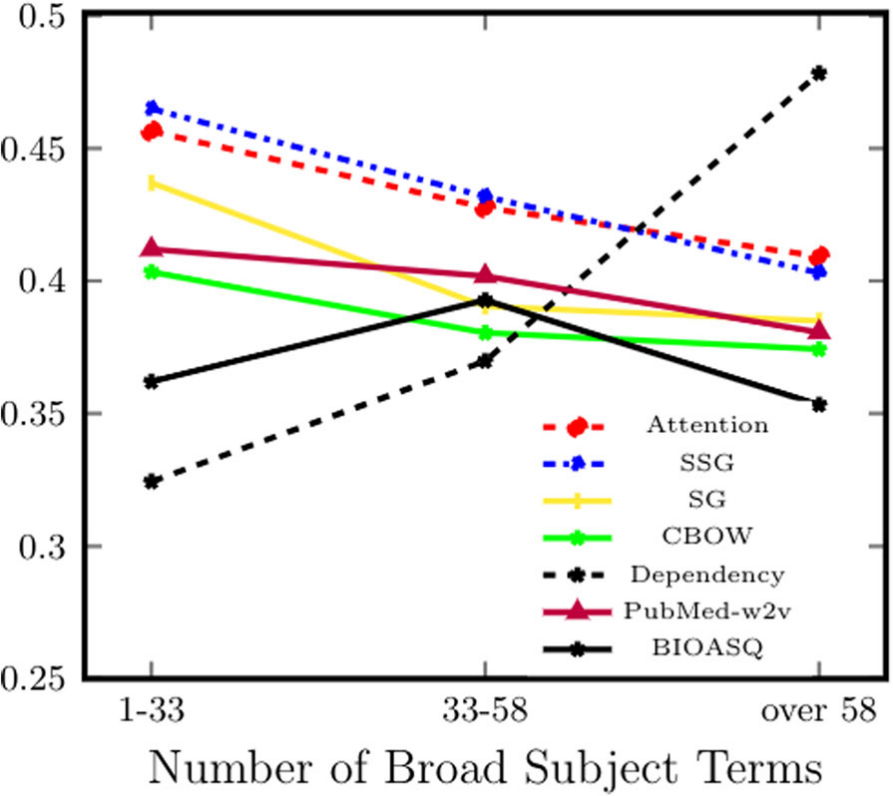
Subset-based evaluation for Bio-SimVerb (y axis unit: *ρ*), where subsets are created based on the word’s number of unique Broad Subject Terms. A word can have multiple Broad Subject terms when it appears in journals of different areas in biomedicine. To be included in each group, it is required that both words in a pair are contained in the same Subject Term interval (x axis)

**Fig. 4:**
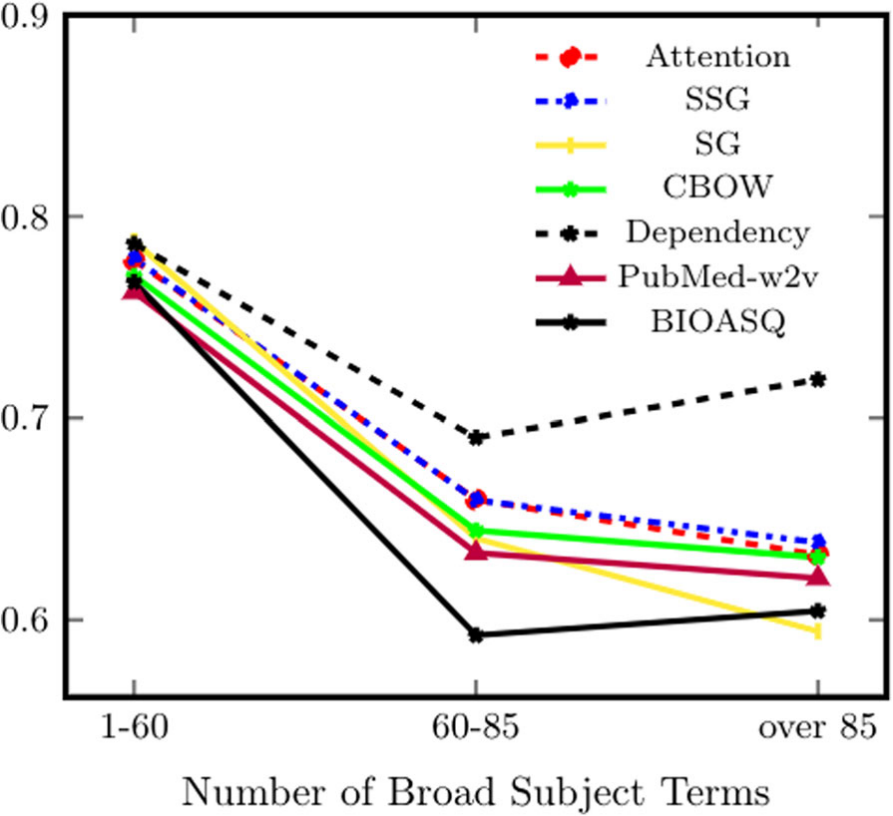
Subset-based evaluation for Bio-SimLex (y axis unit: *ρ*), where subsets are created based on the word’s number of unique Broad Subject Terms. A word can have multiple Broad Subject terms when it appears in journals of different areas in biomedicine. To be included in each group, it is required that both words in a pair are contained in the same Subject Term interval (x axis)

From Figs. 3 and 4, we see a clear overall downward trend, suggesting that it is still a challenge for distributional models to capture the diverse usage patterns of words that appear across different domains. However, using additional information beyond corpus co-occurrence (e.g. dependency parsing) facilitates the learning of representation for such verbs, as reflected in the notable improvement for the dependency model seen in Fig. 3. Intuitively, dependency parses can provide discriminative context to facilitate representation learning: for example, two verbs are similar if they share similar nominal subjects (nsubj and nsubjpass). Nevertheless, our result shows that dependency parses do not contribute equally to the learning of noun and verb representations. Again, this supports our notion that representations of particular word types should be evaluated separately to better understand the type-specific properties learned by different models.

**Human agreement** Since distributional models are sensitive to word-frequency and the diversity of usage patterns, we also examine if these factors affect human perception of word similarity. We report the average standard deviation of ratings per subset in Table 10 (by word frequency) and Table 11 (by Broad Subject Terms). That allows us to compare human agreement across subsets through the ratings of individual items in each subset. In general, the overall average standard deviations across all subsets are almost identical (≈1.0). The subset where we find the highest deviation is the low-frequency subset of Bio-SimLex (Table 10). It is possible that annotators may not have been familiar with some rare words in Bio-SimLex, leading to a higher variance in ratings.

**Table 10:** Average standard deviation of ratings per subset by word-frequency.

Frequency subset	Bio-SimVerb	Bio-SimLex
Low	0.9848	1.5621
Medium	0.8059	0.6784
High	1.2352	1.0237
Average	1.009	1.088

We use: low, medium and high to label subsets for brevity. Range values of corresponding subsets can be found in Figs. 1 and 2

**Table 11:** Average standard deviation of ratings per subset by the number of Broad Subject Term.

Subject subset	Bio-SimVerb	Bio-SimLex
Low	0.8941	1.2395
Medium	0.9084	0.7585
High	1.25	1.1204
Average	1.018	1.039

We use low, medium and high to label subsets for brevity. Range values of corresponding subsets can be found in Figs. 3 and 4

## Conclusions

In this paper, we have presented two novel resources for the evaluation of word representation models: Bio-SimLex and Bio-SimVerb. These datasets allow researchers to investigate how humans and machines represent noun and verb semantics. Their size and coverage of concepts make it possible for the datasets to be used for comparing representation models in different areas of biomedicine. Furthermore, we observe a positive correlation between the performance of biomedical representation models on Bio-SimLex and in biomedical NER. This indicates that our datasets can effectively measure properties that are relevant to performance in extrinsic tasks. We have also examined the impact of different representation learning approaches on nouns and verbs separately, and observed that a single learning approach cannot capture the semantics of all word types. To identify useful methods for learning type-specific representations, resources for the evaluation of individual word types, such as Bio-SimLex and Bio-SimVerb, are indispensable.

### Future work

We observe a positive correlation between the performance of representation models on Bio-SimLex and biomedical NER. It is reasonable to expect that the evaluation of noun representations (Bio-SimLex) is more relevant to performance in NER than evaluation of verb representations (Bio-SimVerb). In the future, we aim to further assess the correlation between performance on Bio-SimVerb and other extrinsic tasks, such as relation typing, where performance is more closely related to the quality of verb representation. To encourage future research in related aspects, we make our datasets available to the community at https://github.com/cambridgeltl/bio-simverb.

## Availability and requirements

**Project Name:** Bio-SimVerb

**Project homepage:** https://github.com/cambridgeltl/bio-simverb

**Operating system:** Mac OS

**Programming language:** Python

## Abbreviations

CBOW: Continuous Bag-Of-Word model
NER: Named entity recognition
NLP: Natural language processing
POS-Tagging: Part of speech tagging
SG: Skip-gram model
